# Development of a Potent Antimicrobial Peptide With Photodynamic Activity

**DOI:** 10.3389/fmicb.2021.624465

**Published:** 2021-06-01

**Authors:** Di Zhang, Jingyi Chen, Qian Jing, Zheng Chen, Azeem Ullah, Longguang Jiang, Ke Zheng, Cai Yuan, Mingdong Huang

**Affiliations:** ^1^College of Chemistry, Fuzhou University, Fuzhou, China; ^2^College of Biological Science and Engineering, Fuzhou University, Fuzhou, China

**Keywords:** antimicrobial peptide, Gram-negative bacteria, phthalocyanine, hydrophobicity, antibiotic resistance

## Abstract

The emergence of antibiotic-resistant bacteria poses a serious challenge to medical practice worldwide. A small peptide with sequence RWRWRW was previously identified as a core antimicrobial peptide with limited antimicrobial spectrum to bacteria, especially Gram-positive bacteria. By conjugating this peptide and its analogs with lipophilic phthalocyanine (Pc), we identified a new antibiotic peptide [PcG_3_K_5_(RW)_3_]. The peptide demonstrates increased antimicrobial effect to both Gram-positive *Staphylococcus aureus* and Gram-negative *Escherichia coli*. In addition, Pc also provides added and potent antimicrobial effect upon red light illumination. The inhibitory efficacy of PcG_3_K_5_(RW)_3_ was increased by ~140-fold to nanomolar range upon illumination. Moreover, PcG_3_K_5_(RW)_3_ was safe for mammalian cell and promoted wound healing in the mouse infection model. Our work provides a new direction to optimize antimicrobial peptides to enhance antimicrobial efficacy.

## Introduction

During the past few decades, antimicrobial resistance (AMR) of bacteria has become a worldwide threat to public health. The WHO has recently issued a priority list of pathogen strains that have acquired resistance to most, and in some cases to all, antibiotics at a global level and advocates urgent needs to develop treatment for these AMR strains for human health (Tacconelli et al., 2018). The list includes both Gram-negative and Gram-positive bacterial strains. Gram-negative bacteria, with an outer membrane to protect themselves from unwanted compounds, are difficult to inhibit by traditional antibiotics (Payne et al., 2007; Lewis, 2013). Daptomycin is a lipopeptide antibiotic approved by the US Food and Drug Administration (FDA) in 2003 to treat infection induced by Gram-positive bacteria (Sauermann et al., 2008). Daptomycin contains amphiphilic 13mer peptide and a decanoyl fatty acid tail. Darobactin is a recently reported peptide antibiotic (Imai et al., 2019) with low toxicity and strong potency against AMR Gram-negative pathogens. Broad-spectrum antibiotics with inhibitory activities to both Gram-positive and Gram-negative strains have strong advantages and are much needed.

Antimicrobial peptides (AMPs) have multiple targets on pathogenic microbes, including the cytoplasmic membrane and cell division and synthesis of essential proteins (Okorochenkov et al., 2012). One drawback of AMPs is their limited antimicrobial spectrum, susceptibility toward proteolytic degradation, and moderate antimicrobial activity (Habets and Brockhurst, 2012; Niu et al., 2013). In addition, the strategy for AMP optimization is generally not well defined, except the development of various formulation methods (Mahlapuu et al., 2016), including nanocarriers. Arginine- and tryptophan-rich peptides are a class of promising lead structures with inhibitory effect against Gram-positive bacterial strains but less activity against Gram-negative bacteria (Strom et al., 2002, 2003; Choi and Moon, 2009; Dennison et al., 2009; Albada et al., 2012; Arias et al., 2014; Wenzel et al., 2014, 2016a,b). The presence of lipophilic groups in AMPs appears to be important for high potency, as demonstrated by two successful examples. Daptomycin is a 13mer lipopeptide antibiotic containing decanoyl fatty acid tail and was approved by the US FDA in 2003 to treat infections caused by Gram-positive bacteria (Sauermann et al., 2008). Darobactin is a recently reported peptide antibiotic (Imai et al., 2019) with low toxicity and strong potency and contains three aromatic rings.

**Scheme 1:**
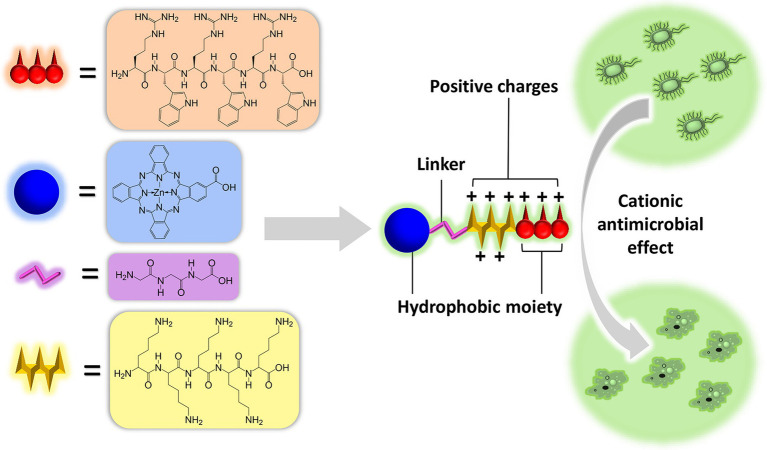
A new antibiotic peptide [PcG_3_K_5_(RW)_3_] was identified by conjugating a small peptide of sequence RWRWRW or its analogues with lipophilic phthalocyanine (Pc). The peptide demonstrates enhanced antimicrobial effect to both Gram-positive *S. aureus* and Gram-negative *E. coli*. In addition, the inhibitory efficacy of the peptide was increased by ~140-fold to nanomolar range upon LED illumination. Moreover, the peptide was safe to mammalian cell, and promoted wound healing in mice infection model.

A hexapeptide RWRWRW was shown to be the shortest unit possessing effective antimicrobial efficacy (Strom et al., 2003; Liu et al., 2007; Albada et al., 2012; Wenzel et al., 2016a). The peptide showed a limited antimicrobial spectrum of only medium antimicrobial activity against Gram-positive bacteria but less activity against Gram-negative bacteria. The structure and functional studies on these arginine- and tryptophan-rich peptides clearly demonstrated the importance of strong lipophobicity (Liu et al., 2007; Albada et al., 2012; Wenzel et al., 2016a) for antimicrobial activity. For example, tyrosine was found to be less effective in providing bulk and lipophilicity than tryptophan (Strom et al., 2003). In this work, we incorporate a new lipophilic group, phthalocyanine (Pc), into the RWRWRW unit to synthesize a series of peptides and identify a potent AMP (Scheme 1). Pc not only provides hydrophobicity for bacterial membrane attachment but also becomes photocytotoxic to microbes when illuminated by red light at a specific wavelength [680 nm; Morley and Charlton, 1995; Gardberg et al., 2001; named antimicrobial photodynamic therapy (aPDT)]. No known bacterial resistance to aPDT has been reported (Chen et al., 2017; Wainwright et al., 2017). The peptide is shown to be safe to mammalian cells and has antimicrobial activity in a wound infection mouse model. Importantly, the peptide is active not only against Gram-positive *Staphylococcus aureus* but also against Gram-negative bacteria and even to multiresistant bacterial strains [methicillin-resistant *S. aureus* (MRSA)]. In addition, with the illumination of red light, the antimicrobial efficacy increased by ~140-fold against either Gram-positive or -negative bacterial strains (IC_50_s of 85 nM against *S. aureus* and 163 nM against *Escherichia coli*). We also demonstrated the antibacterial efficacy of the peptide in a mouse skin infection model. The strategy used here can be used to empower and expand the antimicrobial spectrum of other AMPs.

## Materials and Methods

### Materials

The side chain-protected peptides G_3_(RW)_3_, G_3_(RW)_3_K_5_, and G_3_K_5_(RW)_3_ on Wang resin were obtained commercially (GL Biochem Ltd., Shanghai). Beta-carboxy phthalocyanine zinc was developed by us by a method previously reported (Chen et al., 2006). Beta-carboxy phthalocyanine (β-PcCOOH) was purified on a silica column with N,N-dimethylformamide (DMF):acetone (3:1) as the second band. All the chemical reagents were analytical grade and used without further purification. Distilled water was used throughout the experiments. *E. coli* (ATCC 8739), *S. aureus* (ATCC 6538), and MRSA (ATCC 33591) were acquired from ATCC. Bioluminescent strains of *E. coli* DH5α were constructed by transformation of plasmid pAKlux2.1, which contained a complete bacterial luciferase operon as described (Ullah et al., 2018; Chen et al., 2019), and *S. aureus* Xen29 (NCTC8532) containing a stable copy of the modified Photorhabdus luminescens luxABCDE operon was purchased from Shanghai Biofeng Company.

### Synthesis and Characterization

β-PcCOOH was conjugated with side chain-protected G_3_(RW)_3_, G_3_(RW)_3_K_5_, or G_3_K_5_(RW)_3_ on Wang resin. In a typical conjugation reaction, β-carboxy phthalocyanine zinc (49.76 mg, 0.08 mmol) was dissolved in 2 ml DMF. Hexafluorophosphate benzotriazole tetramethyl uronium (HBTU; 61 mg, 0.16 mmol) and diisopropylethylamine (DIEA; 0.1 ml) were added to the solution and stirred for 30 min. The Wang resin with peptide G_3_(RW)_3_, G_3_(RW)_3_K_5_, or G_3_K_5_(RW)_3_ (200 mg, 0.08 mmol) was added into the solution, and the mixture was stirred for 24 h. After the reaction was completed, the filtered solid was washed three times by DMF and methanol until the filtrate became colorless. The residue was then treated with 95% trifluoroacetic acid (TFA) for 4 h at room temperature to remove the protecting groups and the Wang resin. The TFA-treated solution was filtered on a Buchner funnel, and the filtrate was concentrated with rotary evaporation, followed by the addition of cool anhydrous ethyl ether to obtain precipitate. The new synthetic compounds were further purified on a preparative high-performance liquid chromatography (HPLC; Dalian Elite Analytical Instruments Co. Ltd., Dalian, China) using a reverse phase column (Sino Chrom ODS-BP, 10 mm), eluted with a linear gradient of 50–100% in dimethyl sulfoxide (DMSO) in 0.01% TFA in a period of 30 min at a flow rate of 5 ml/min. UV-Vis spectra and photo-degraded curve was measured by BioTek Synergy 4 multi-mode microplate reader in a 96-well plate at room temperature. The UV-Vis absorption spectrum of antimicrobial Pc-peptides (APPs) in DMSO was typical of Pc with the strongest absorption at 678 nm (extinction coefficient of 118,380 L·mol^−1^·cm^−1^). The synthetic AMPs were further characterized by high-resolution mass spectra.

### Antimicrobial Activity Assays Using Bioluminescent Bacteria

Bioluminescent bacteria were grown in Luria-Bertani (LB) culture medium at 37°C under aerobic conditions overnight 8–12 diluted 100 times to 10^6^ colony-forming units (CFU)/ml in phosphate buffered saline (PBS) before incubating with the peptides. The peptide or new compounds were first dissolved in PBST (PBS with 0.05% Tween 20) to prepare 1-mM stock solutions, and the solutions were then further diluted up to 100 μM with PBST. The bacterial suspensions were then incubated in 96-well plates with the compound peptides. The luminescence was monitored for 1 h to measure the bacterial viability. The percentage of survived bacteria was determined from the luminescence intensities of the treated group divided by the luminescence signal of the controls.

For photo-assisted antimicrobial activity measurements against bioluminescent bacteria, we used a procedure similar to as above, but with illumination using LM-LED light (680 nm) for 6 min (light dose of 12 J/cm^2^, 33.33 mW/cm^2^) after 10 min incubation of the bacterial suspensions and the compound peptides.

### Antimicrobial Activity Assays Using ATCC Standard Strains

*E. coli* (ATCC 8739), *S. aureus* (ATCC 6538), or MRSA (ATCC 33591) were grown in LB culture medium at 37°C under aerobic conditions overnight (8–12 h) before use.

Minimum inhibitory concentrations (MICs) were measured by the double dilution method. Inocula of bacteria were prepared by adjusting overnight cultures in LB medium. Aliquots of 100 μl of the inocula were mixed with 900 μl peptides of serial double dilutions in centrifuge tubes and then incubated with shaking at 37°C for 20 h without light. MICs were defined as the lowest concentrations of the peptides that completely inhibit the growth of bacteria (final concentration of 10^6^ CFU/ml). Bactericidal kinetics assay was also determined. Different final concentrations (0, 1/8-, 1/4-, 1/2-, 1-MIC) of the peptides were added to the bioluminescent *S. aureus* suspension (10^3^ CFU/ml). The bacteria were incubated at 37°C and 150 rpm. An aliquot of 100 μl of the suspension was taken out to measure the absorption at 600 nm on microplate reader once an hour for up to 12 h.

Photo-assisted antimicrobial activity assays of the compound peptides against ATCC standard strains were carried out by colony counting method. The agar plates with only bacterial suspension were used as negative control. The peptide and bacterial suspension were incubated together in 96-well microplates first. Under the same conditions, for comparison, the other groups were set without light. After incubation for 40 min at room temperature, aliquots of 100 μl were taken out of the solutions and placed on agar plates at serial dilutions, then incubated at 37°C for 18–24 h. Numbers of colonies were counted, and the antimicrobial rate (R) was calculated: R = (B-A)/B × 100%. Here, A is the bacterial colony number on the agar plates containing various concentrations of the peptides, and B is the colony number for negative control. A similar procedure was carried out to study the effect of low dose of light on the activity of PcG_3_K_5_(RW)_3_ against *S. aureus* (ATCC 6538) and *E. coli* (ATCC 8739), except the changing irradiate light doses (0, 0.15, 0.3, 0.45, and 0.6 J/cm^2^).

### Reactive Oxygen Species Measurement

The reactive oxygen species (ROS) generated by Pc mainly includes singlet oxygen and may also contain hydroxyl radical in some cases. In this study, the ROS generation of PcG_3_K_5_(RW)_3_ was measured with a probe [2,7-dichlorofluorescein diacetate (DCFH-DA)]. In the presence of ROS, 2',7'-dichlorofluorescin diacetate (DCFH-DA) can be converted to 2,7-dichlorofluorescein (DCF, ex 400 nm, em 528 nm). 1,3-Dimethyl-2-thiourea (DMT) is a quencher of hydroxyl radical, while NaN_3_ is a quencher for singlet oxygen. In a 96-well white plate, peptide PcG_3_K_5_(RW)_3_ or G_3_K_5_(RW)_3_ (for control) was added into the solution of DCFH-DA (100 μM) with or without DMT (1 M) and/or NaN_3_ (30 mM) to the final concentration of peptide at 10 μM and the total volume of 200 μl (using PBS as buffer). The solutions were illuminated using a planar LED light source for 5 min at intervals, and the emission intensity of DCF at 528 nm was measured every 30 s after illumination. The no-light radiation group was used as control.

### Scanning Electron Microscope

The morphology changes of bacteria treated with PcG_3_K_5_(RW)_3_ were observed using SEM. Untreated bacteria acted as the control. *E. coli* strains (ATCC 8739) were harvested by centrifuging at 6,000 rpm for 10 min and washed with sterile PBStwice. For the fixation, the bacteria were fixed with precooling 2.5% (v/v) glutaraldehyde in PBS overnight at 4°C. For dehydration, the bacteria were dehydrated by a graded series of ethanol (30, 50, 70, 90, and 100%) for about 10 min. The dehydrated *E. coli* solution was dropwise onto silicon wafer and dried at 37°C overnight. Then, prepared specimens were sprayed with gold before observation using SEM.

### Bacterial Membrane Permeability Assay

Membrane permeability of bacteria was determined by the 8-aniline-1-naphthalene sulfonic acid (ANS) uptake assay. The bacteria (10^8^ CFU/ml) were washed with PBS and suspended (3,000–4,000 rpm) to OD_600_ = 0.1–0.3. PcG_3_K_5_(RW)_3_ (final concentration of 10 μM) was added to bacterial suspension (*E. coli* ATCC 8739) before illumination (light dose of 12 J/cm^2^, 33.33 mW/cm^2^) was conducted. A group without light illumination was set up as control. The fluorescent hydrophobic probe ANS with a final concentration of 5.65 mM was added to bacterial suspension, and fluorescence intensity was recorded. The excitation and emission wavelengths of ANS were set at 380 and 520 nm, respectively.

### *In vivo* Antimicrobial Test in Mammal Anti-infection Model

A localized infection mouse model was established to evaluate the antibacterial activity of PcG_3_K_5_(RW)_3_ against *S. aureus in vivo* according to the procedure that we previously established (Zhang et al., 2018; Chen et al., 2019). Kunming mice (4-week-old, 25 ± 2 g, purchased from Shanghai SLAC Laboratory Animal Co. Ltd., Shanghai, China) were maintained and handled in accordance with the recommendations of the Institutional Animal Care And Use Committee (IACUC). Mice were divided into three groups: two groups were treated with PcG_3_K_5_(RW)_3_ and *S. aureus*, with or without light, another group was treated with only *S. aureus*. Each group had six mice. All groups of mice were allowed free access to water and food throughout the experimental process. In each group, excisional wounds (10 mm × 10 mm) was made on the dorsal surface of the mouse by disinfected scissors to a depth of 2.0 mm. The bottom of the wound was panniculus carnosus with no visible bleeding. An aliquot (50 μl) of mid-log phase *S. aureus* (10^8^ CFU/ml) was then inoculated into each wound of mice. In the experimental group, 50 μl saline solution of PcG_3_K_5_(RW)_3_ at final concentration of 20 μM (concentration of MIC) was added into the wound surface after inoculation with *S. aureus*. The area of the wounds and body weight of mice were measured each day.

### *In vivo* Biosafety and Stability Measurement

In order to evaluate the biosafety of PcG_3_K_5_(RW)_3_, we measured its cytotoxicity on human embryonic lung fibroblast cells. Aliquots (100 μl) of cells (∼10^5^ per ml) were incubated in 96-well plates with PcG_3_K_5_(RW)_3_ at various concentrations (0.8, 1.6, 3.1, 6.3, 12.5, 25, 50, and 100 μM) at 37°C for 24 h. The culture medium was used as control. The cells were washed with PBS, then were divided into two groups, and the light group was exposed to 12 J/cm^2^ of 680 nm light illumination. After another 12-h incubation, the culture medium was refreshed and 3-(4,5-dimethylthiazol-2-yl)-2,5-diphenyltetrazolium bromide (MTT) was added. After 4-h incubation at 37°C, the absorbance at 490 nm was measured on a multi-mode microplate reader (BioTek Synergy 4). The inhibition rate of cell growth was calculated by the following equation:

$$\mathrm{Cell viability}\;\left(\%\right)=\left(\frac{\begin{array}{l} Mean\;absorbance\;value\;of\;\\ treatment\;group \end{array}}{\begin{array}{l} Mean\;absorbance\;value\;of\;\\ control \end{array}}\right)\times 100\%$$

Meanwhile, an *in vitro* hemolysis assay was performed to evaluate hemoglobin (Hb) release in the plasma (as an indicator of red blood cell lysis) following peptide PcG_3_K_5_(RW)_3_ exposure. Typically, 450 μl 0.9% NaCl, 450 μl 2% red blood cells, and 100 μl PcG_3_K_5_(RW)_3_ (at final concentrations of 0.5, 5, 50, and 500 μM) were mixed together and incubated in a 37°C for 30 min. Positive control (containing 450 μl 2% red blood cell only) and negative control (containing 550 μl 0.9% NaCl and 450 μl 2% red blood cell) were designed as quality controls. Then, red blood cells were pelleted down by centrifuging the samples at 1,500 rpm for 10min. The supernatant was aspirated, and the extent of hemolysis was quantified by determining the amounts of released Hb in the supernatant at 545 nm against Hb standard. Hemolysis rate *Z* can be calculated by the following equation:

$$Z\;\left(\%\right)=\left(\frac{Dt-Dnc}{Dpc-Dnc}\right)\times 100\%$$

Here, *Dt*, *Dnc*, and *Dpc* represent the absorption values at 545 nm of samples, the negative controls, and the positive controls.

In order to test the stability of PcG_3_K_5_(RW)_3_ when exposed to some proteases in human, we incubated the saline solution of PcG_3_K_5_(RW)_3_ (final concentration of 100 μM) with high concentration (200 nM) of trypsin and equal volume of saline solution for the control group. Then, all groups were incubated at 37°C for 30 min. Supernatant of centrifugation was measured in HPLC, and the procedure was the same as we mentioned above.

### *In vivo* Biodistribution and Clearance Measurement

In order to find out metabolic characteristics of PcG_3_K_5_(RW)_3_ *in vivo*, we evaluated the biodistribution and clearance of PcG_3_K_5_(RW)_3_ in organs and blood of mice. Kunming mice were divided into eight groups (five mice per group), and the PcG_3_K_5_(RW)_3_ (50 μM), RWRWRW (0.4 mg/kg of mouse body weight), or saline was injected through the tail vein. The mice were sacrificed at 2, 4, 8, 12, 24, 36, 48, and 72 h post-injection, and their primary organs (liver, kidneys, heart, spleen, lung, stomach, intestines) or muscles were collected postmortem. Meanwhile, their blood was collected through enucleation of eyeballs. These samples were then washed with saline and imaged on the three-dimension FMT 2500TM LX (PerkinElmer, Waltham, MA, United States) using the same acquisition settings as the *in vivo* imaging to quantitate the peptide average concentrations in the samples.

The blood of mice was diluted 10 times with DMF, immediately after collection, and then centrifuged to get supernatant for further determination. The supernatant was measured by fluorescence spectrophotometer F-4600 (HITACHI, Tokyo, Japan). The fluorescence intensity was recorded. The excitation and emission wavelengths were set at 610 and 680 nm. Then, the plasma concentrations of the PcG_3_K_5_(RW)_3_ were determined using a concentration-fluorescence standard curve we made. The plasma half-life (*t*_1/2_) can be calculated by the following formula:

$$t_{1/2}=\frac{0.693}{\left(-2.303k\right)}$$

Here, *k* represents the slope of fluorescence intensity-log (concentration) regression equation.

### Statistical Analysis

Each test was repeated at least three times. The data were presented as the means ± SD. Statistical analysis was performed using one-way analysis of variance. Multiple comparisons of the means were done by the least significant difference test. All computations were made by employing statistical software.

## Results and Discussion

### New Antimicrobial Peptides: Design, Synthesis, and Characterization

Introduction of hydrophobic moieties, like acyl or aliphatic chains, is an optimization strategy for stronger antimicrobial efficacy and broader antimicrobial spectrum (Radzishevsky et al., 2005, 2007; Smith et al., 2018). Pc is a synthetic hydrophobic compound with aromatic ring size equivalent to four indole side chains of tryptophan. The use of Pc has an added feature: upon absorption of light at 680 nm (maximal absorption of the Pc), Pc will undergo inter-system crossing and react with molecular oxygen, generating ROS that eliminates pathogens nearby (Zhang et al., 2018; Chen et al., 2019).

**Figure 1:**
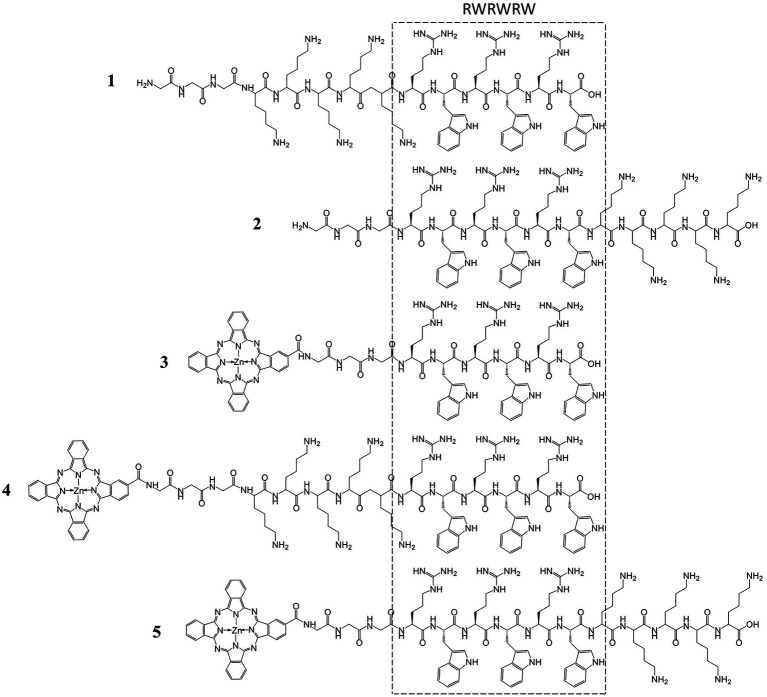
Designed structures of antimicrobial Pc-peptides based on core peptide RWRWRW_3_.

Here, we design a series of AMPs with Pc linked to RWRWRW hexapeptidyl unit separated by a triglycine spacer (Figure 1). The carboxyl terminal of peptides was not amidated to promote aqueous solubility after conjugating hydrophobic Pc group. Pentalysine moiety (K_5_) was also integrated into the peptide chain for two purposes: (1) enhance aqueous solubility and (2) increase antimicrobial efficacy to Gram-positive strains. Polylysine is an FDA-approved food preservative used in sushi, with a dose up to 50 mg/kg, and has a wide antimicrobial spectrum with good activity inhibiting Gram-negative bacteria. Here, the pentalysine was incorporated at two different orientations: either before or after RWRWRW unit. As controls, the peptides without the Pc group or pentalysine moiety (K_5_) were also generated for comparison.

**Figure 2:**
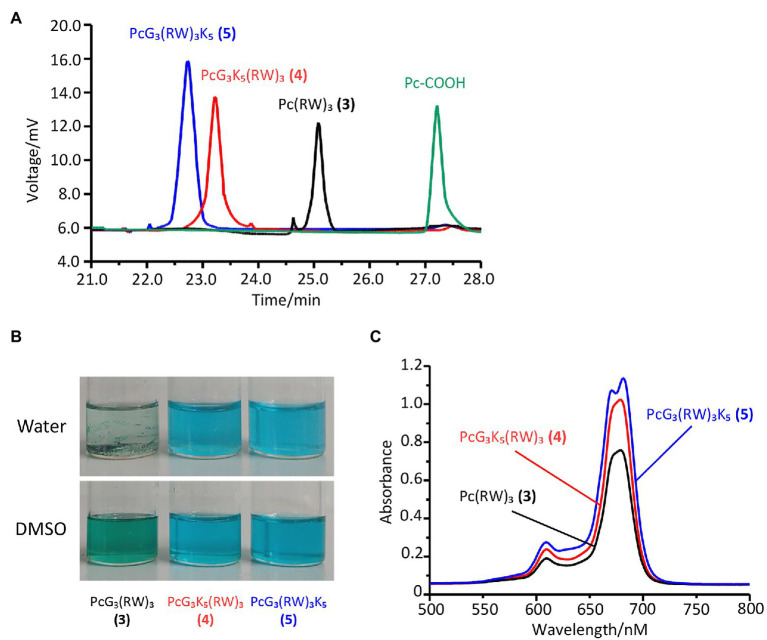
Characterization of the antimicrobial Pc-peptides (APPs) and Pc-COOH. The APPs had high purity based on high-performance liquid chromatography (HPLC) profiles on C18 column **(A)** and different solubility in both dimethyl sulfoxide (DMSO) and water **(B)**. All APPs exhibited a strong absorption band at 678 nm in DMSO **(C)**.

To synthesize these peptides, the side chain-protected peptides were commercially acquired, followed by their covalent conjugation at amino terminal to a mono-carboxy Pc (Chen et al., 2006, 2011; Supplementary Figure S1). The compounds were purified to highly homogeneous based on C18 reverse phase column on HPLC. The order of retention times of the compounds was consistent with the hydrophilicity and polarity predicted based on their molecular structures (Figure 2A). Compounds **4** and **5** are well soluble in DMSO and aqueous solution. By comparison, compound **3** shows poor solubility in water (Figure 2B). The soluble antimicrobial Pc-peptide (APP) compounds **4** and **5** were characterized by electrospray ionization (ESI)-mass spectrometry (MS; Supplementary Figure S2). All the synthetic antimicrobial Pc-peptides (APPs) showed strong absorptions at 680 nm in DMSO (Figure 2C), the typical feature of Pc (Chen et al., 2006).

### Antimicrobial Activities of the Antimicrobial Pc-Peptides *in vitro*

To evaluate the antimicrobial activity of APPs, we used bioluminescent Gram-positive *S. aureus* and Gram-negative *E. coli*, where the bioluminescent intensity [relative luminescence units (RLUs)] was proportional to the number of live bacteria. The peptides at various concentrations were incubated with the strains, and the luminescence was monitored on a microplate reader to obtain the IC_50_ of new peptides against these bacteria (Table 1). Compound **4** or **5** was shown to possess 2–4 times higher efficacy on both bacterial strains than compound **1** or **2** (Table 1), especially against Gram-negative *E. coli*, which was likely due to the extra hydrophobicity provided by the Pc group. Compound **3** [PcG_3_(RW)_3_] exhibited moderate efficacy to bacteria compared to compound **4** or **5** but with poor aqueous solubility. Compound **4** [PcG_3_K_5_(RW)_3_] turned out to be the most effective compound against either Gram-positive *S. aureus* or Gram-negative *E. coli* with IC_50_s of 12.4 and 23.9 μM, respectively. It is likely that both Pc moiety and tryptophan residue of compound **4** anchor directly into the phospholipid bilayer of bacterial membrane and further lead to cell death.

**Table 1:** Half-maximal inhibitory concentrations (IC_50_s) of antimicrobial peptides against bacteria.

Compd #, peptide sequence	IC_50_ (μM) against	
	*S. aureus*	*E. coli*
1, G_3_K_5_(RW)_3_	45.6 ± 2.9	61.8 ± 4.3
2, G_3_(RW)_3_K_5_	68.0 ± 3.2	135 ± 25.3
3, PcG_3_(RW)_3_	34.0 ± 4.0	73.8 ± 1.7
4, PcG_3_K_5_(RW)_3_	12.4 ± 0.9	23.9 ± 1.0
5, PcG_3_(RW)_3_K_5_	34.7 ± 3.1	51.2 ± 5.5

Half-maximal inhibitory concentrations (IC_50_s) of antimicrobial peptides against Gram-positive strain *S. aureus* or Gram-negative strain *E. coli*. Each experiment was performed in triplicate.

To further validate the antimicrobial efficacy, we measured MIC on two bacterial strains (*E. coli* ATCC 8739, *S. aureus* ATCC 6538) and the MRSA strain (Table 2). The results verified the conclusion obtained using bioluminescent bacteria. Compounds with Pc group (compounds **3**, **4**, and **5**) exhibited much stronger inhibitory efficacies against either *E. coli* or *S. aureus* than the compounds of peptides. Furthermore, MRSA strain showed similar sensitivity to the peptides as the wild-type *S. aureus* strain. It should be mentioned that the antimicrobial efficacy data here are in the same range but slightly lower than those previously reported for RWRWRW-NH_2_ (Strom et al., 2002, 2003; Liu et al., 2007; Albada et al., 2012). This is likely due to the lack of C-terminal amidation of our peptides, which appears to be a key parameter for the efficacy.

**Table 2:** Minimum inhibitory concentrations (MICs) of antimicrobial peptides against different types of bacterial strains.

Compd #, peptide sequence	Molecular mass	MIC (μM) against		
		*S. aureus*	MRSA	*E. coli*
1, G_3_K_5_(RW)_3_	1,855.1	107.8	107.8	>215.6
2, G_3_(RW)_3_K_5_	1,855.1	107.8	107.8	>215.6
3, PcG_3_(RW)_3_	1,676.7	12.0	24.0	48.0
4, PcG_3_(RW)_3_K_5_	2,488.2	8.0	16.1	16.1
5, PcG_3_K_5_(RW)_3_	2,488.2	16.1	16.1	16.1

Each experiment was performed in triplicate.

**Figure 3:**
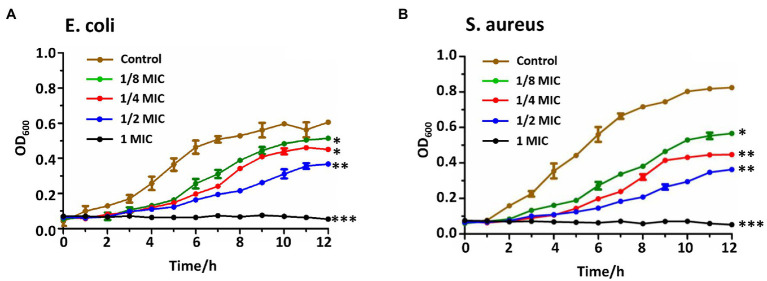
Bactericidal kinetics of PcG_3_K_5_(RW)_3_. The bacterial growth of *Escherichia coli* **(A)** and *Staphylococcus aureus* **(B)** were slowed down in the presence of different concentrations at the concentration of PcG_3_K_5_(RW)_3_ at 1/8, 1/4, 1/2, 1-fold of minimum inhibitory concentration (MIC). Each experiment was performed in triplicate. *, **, and *** indicate significant differences (*p* < 0.05, *p* < 0.01, and *p* < 0.001) from the corresponding control group.

In addition, the bactericidal kinetic curves (Figure 3) were recorded to test the continuous antimicrobial effect on *S. aureus* and *E. coli* bacterial strains in 12 h with varied concentrations (0-, 1/8-, 1/4-, 1/2-, 1-MIC) of compound **4**, which exhibited the strongest activity among all the APPs. The bacteria grew the fastest in the absence of compound **4** and reached the highest plateau among this series. The bacterial growth rate decreased in proportion to the increased concentrations of compound **4**. At the concentration of MIC, the bacterial proliferation completely stopped (Figure 3).

### Photo-Assisted Toxicity of the Antimicrobial Pc-Peptides *in vitro*

**Figure 4:**
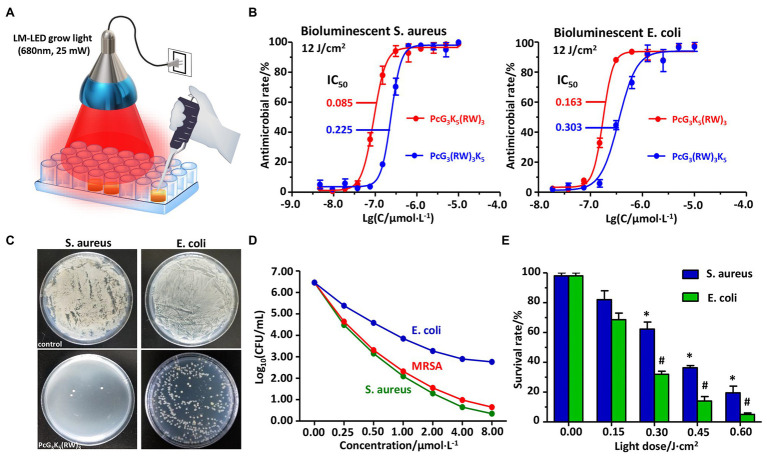
The antimicrobial activity of PcG_3_K_5_(RW)_3_ (4) in the presence of light. The efficacy was measured in 96-well microplates under illumination by LM-LED **(A)**. PcG_3_K_5_(RW)_3_ showed higher antimicrobial activity against both bioluminescent *S. aureus* and bioluminescent *E. coli* under illumination (light dose of 12 J/cm^2^, 33.33 mW/cm^2^) at a low concentration (2 μM) compared to PcG_3_(RW)_3_K_5_ **(B)**. PcG_3_K_5_(RW)_3_ induced up to 5-log reduction of *S. aureus* and 3-log reduction of *E. coli* under illumination (light dose of 12 J/cm^2^, 33.33 mW/cm^2^) at a low concentration (2 μM), which were measured by colony counting method **(C)**. This peptide also showed similar activity against multiple resistant strains [methicillin-resistant *S. aureus* (MRSA)] **(D)**. The result of short illumination indicated that the high light dose was not essential to trigger bacterial cell death **(E)**. Error bars indicated SD for three replications. * and # indicate significant differences to the controls of *S. aureus* (light dose = 0) and *E. coli* (light dose = 0), respectively (*p* < 0.05).

The large aromatic ring Pc also serves as a photosensitizer, generating singlet oxygen when illuminated with a 680-nm light, leading to an additional level of toxicity to bacteria (Ali-Adib et al., 1998; Wrobel and Dudkowiak, 2006; Bian and Jiang, 2016). We used a LED light source that provides a stable and uniform light with the maximal intensity around 680 nm without heating the sample (Figure 4A). We measured the IC_50_ of compounds **4** and **5** on bioluminescent bacteria at a light dose of 12 J/cm^2^ (Figure 4B). The photo-assisted antimicrobial toxicity of this series of compounds had an overall trend similar to the toxicity without light illumination (Tables 1 and 3). Compound **4** was found to have a strong antimicrobial efficacy, with IC_50_s of 85 and 163 nM toward *S. aureus* and *E. coli*, respectively. We also measured the photo-assisted antimicrobial efficacy by colony counting method using ATCC standard strains at a starting concentration of 10^6^ CFU/ml (Figure 4C). The results showed that compound **4** induced up to 5-log (i.e., 99.999%) reduction of *S. aureus* (including antibiotic-resistant strain MRSA) and 3-log (i.e., 99.9%) reduction of *E. coli* at a low concentration (2 μM) with 6 min illumination (light dose of 12 J/cm^2^, 33.33 mW/cm^2^; Figure 4D). We also studied the effect of light dose for PcG_3_K_5_(RW)_3_ and found even a low dose of light (36-s exposure or 0.60 J/cm^2^, 33.33 mW/cm^2^) led to the elimination of about 80% of *E. coli* or 95% of *S. aureus* at the concentration of 2 μM (Figure 4E).

**Table 3:** IC_50_s of antimicrobial Pc-peptides upon illumination (12 J/cm^2^, 33.33 mW/cm^2^).

Compd #, peptide sequence	IC_50_ (μM) against	
	*S. aureus*	*E. coli*
1, G_3_K_5_(RW)_3_	44.640 ± 4.720	59.240 ± 1.970
2, G_3_(RW)_3_K_5_	71.580 ± 2.820	129.230 ± 30.340
3, PcG_3_(RW)_3_	0.108 ± 0.029	0.252 ± 0.014
4, PcG_3_K_5_(RW)_3_	0.085 ± 0.020	0.163 ± 0.018
5, PcG_3_(RW)_3_K_5_	0.225 ± 0.056	0.303 ± 0.036

Half-maximal inhibitory concentrations (IC_50_s) of antimicrobial peptides against Gram-positive strain *S. aureus* or Gram-negative strain *E. coli* when exposed to LED light (25 mW) for 6 min. Antimicrobial activity of Pc-peptides was further enhanced. Each experiment was performed in triplicate.

### Action Mechanisms of PcG_3_K_5_(RW)_3_

**Figure 5:**
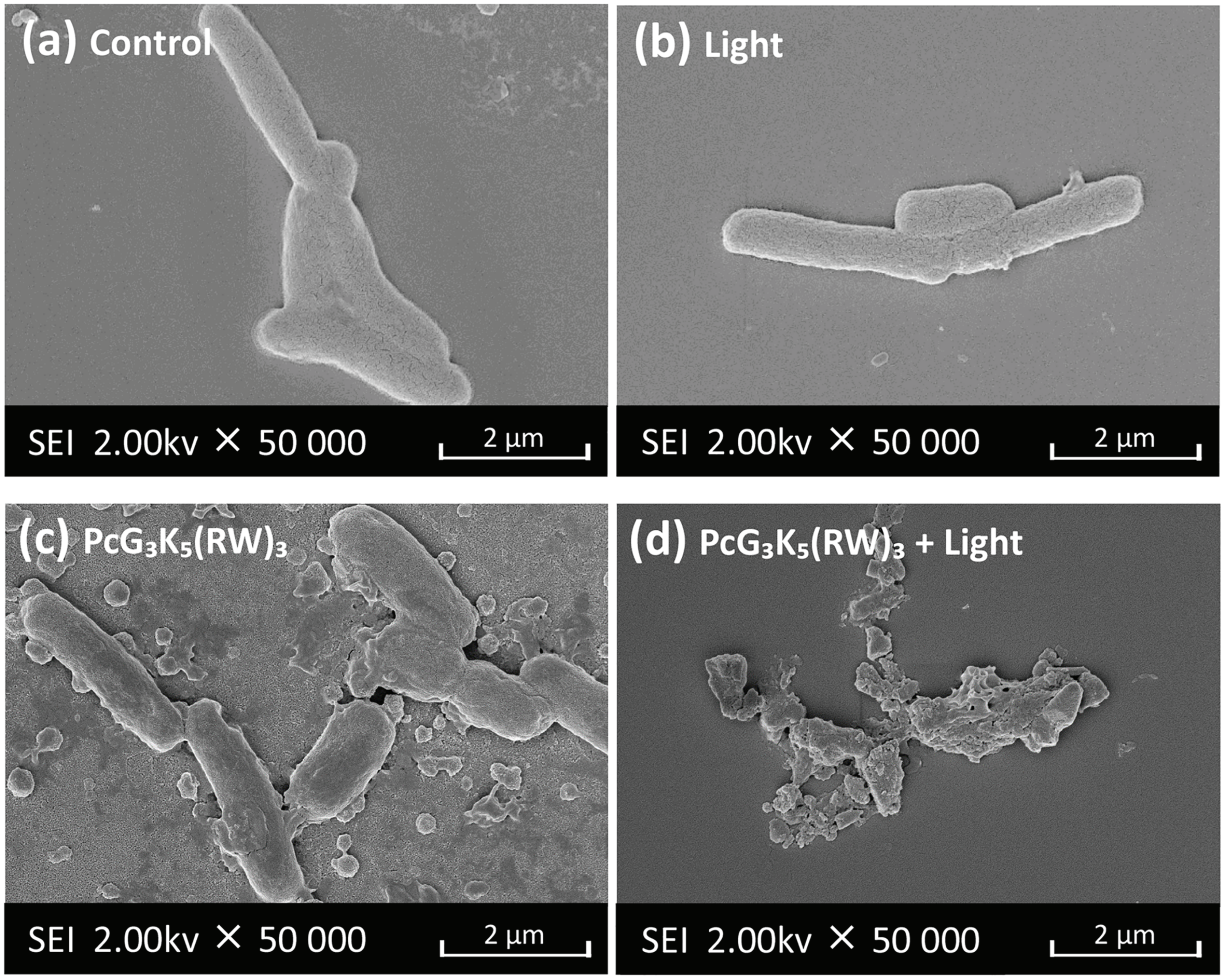
SEM micrographs of *E. coli* after incubation with PcG_3_K_5_(RW)_3_ (23.9 μM, i.e., IC_50_ without light) followed with **(A)** or without light illumination (6 J/cm^2^ 680 nm) **(B)**. *E. coli* group without incubation with PcG_3_K_5_(RW)_3_ **(C)** and *E. coli* group with light exposure **(D)** were used as controls.

The classic antimicrobial mechanism of AMPs is through disruption of the membrane. We studied the changes of bacterial membrane permeability after treatments by ANS, a probe for membrane permeability (Joshi et al., 1988; Liu et al., 2018). Once the probe enters the bacterial phospholipid bilayer membrane, its fluorescence intensity will increase (Misra et al., 2015). This reflects the accumulation of the probe in the lipid bilayer presumably due to the damage of cell wall, allowing more probe penetrating through cell wall and entering bacterial membrane. We observed that our peptide greatly enhanced the ANS fluorescence intensity (Supplementary Figure S3), demonstrating the increased membrane permeability. We also used SEM to study the surface morphology of *E. coli* after incubation with PcG_3_K_5_(RW)_3_ with or without illumination of 680 nm. The results showed that *E. coli*, a bacterium in a rod-like shape with a smooth surface (Figure 5A), presents little changes upon illumination (Figure 5B), while PcG_3_K_5_(RW)_3_ induced morphological damage to *E. coli* (Figure 5C), a bacterium that was typically in a rod-like shape with a smooth surface but became wrinkled and twisted in the presence of PcG_3_K_5_(RW)_3_. The damage was further exacerbated with light illumination (Figure 5D), leading to the outflow of cellular contents and complete fragmentation of the bacterial envelop.

Next, we studied the generation of ROS produced by PcG_3_K_5_(RW)_3_ with 5 min illumination of red light (Supplementary Figure S4; Wainwright et al., 2017; Zhang et al., 2017). We observed the steady increase of fluorescence signals of ROS probe DCFH-DA with time, indicating the production of ROS. Two types of ROS are typically produced by Pc under light illumination: either Type I (free radical) or Type II (singlet oxygen ^1^O_2_), which can be quenched by either DMT or NaN_3_, respectively. We observed that the fluorescence of PcG_3_K_5_(RW)_3_ was quenched to varying degrees in the presence of DMT or NaN_3_ and reached the lowest in the presence of both quenchers. This indicated that PcG_3_K_5_(RW)_3_ generates both types of ROS upon illumination.

### Antimicrobial Activity of PcG_3_K_5_(RW)_3_ *in vivo*

**Figure 6:**
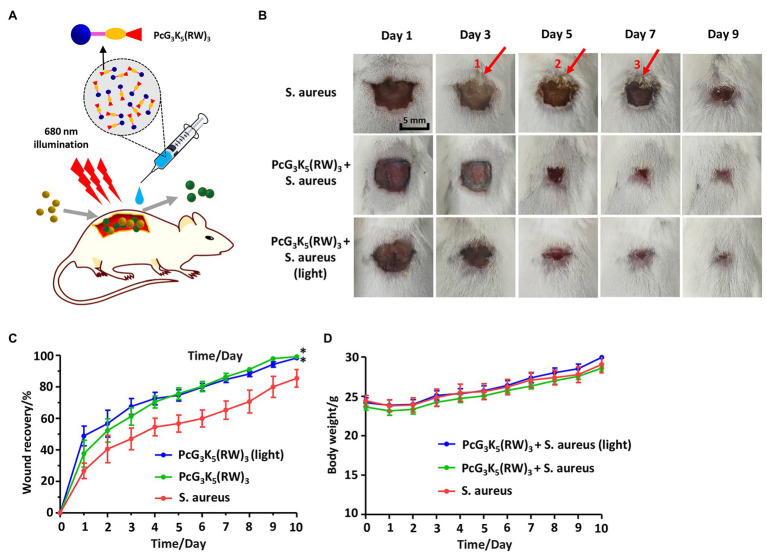
PcG_3_K_5_(RW)_3_ promoted wound healing of bacteria-infected wounds. **(A)** Schematic of animal experiment using PcG_3_K_5_(RW)_3_. **(B)** Images of the wounds in the wound healing model treated in 10 days after surgery. Scale bar: 5 mm (arrow 1 showed the infected area at wound edge, and arrows 2 and 3 showed the scab formed in infected area). **(C)** Effects of PcG_3_K_5_(RW)_3_ (final concentration of 20 μM) on the healing of *S. aureus* treated excisional wounds in mouse (*n* = 6 per group). **(D)** The mice gained body weight constantly, demonstrating safety of the antimicrobial peptide. Error bars indicated SD for three replications. * indicated a significant difference (*p* < 0.05) from the corresponding control group.

*S. aureus* is one of the leading causes of wound infection in hospitals and in the community and causes skin or soft tissue infections, which retard wound healing and may further lead to serious complications. A localized anti-infection model was established to evaluate the antibacterial activity of PcG_3_K_5_(RW)_3_ against *S. aureus in vivo* (Figure 6A). The wounds without antimicrobial compounds showed infection and ulceration at the 3rd day after surgery (Figure 6B). The infection recovered 1–2 days later, and an identifiable scab formed in the infected area. However, the infection did not appear in the groups treated with the local administration of PcG_3_K_5_(RW)_3_. This treatment group showed clear healing on the next day after surgery, indicating rapid anti-infection effects of the compound PcG_3_K_5_(RW)_3_ on traumatic wound (Figure 6C). The results showed that PcG_3_K_5_(RW)_3_ promoted wound healing and prevented or alleviated the infection.

In another group, mice were treated with one-time 680-nm illumination (12.0 J/cm^2^, 33.33 mW/cm^2^) after addition of PcG_3_K_5_(RW)_3_ (Supplementary Figure S5). This group showed better wound recovery than the ones without illumination at the first 3 days after surgery. This verified enhanced of illumination on inhibitory efficacy *in vitro*. Although the light was not essential for PcG_3_K_5_(RW)_3_ to promote wound healing in the mammalian infection model, illumination of low-dosage light (below 12 J/cm^2^, 33.33 mW/cm^2^) is still an additional method to enhance the efficacy in clinical treatment. Besides, treatment with PcG_3_K_5_(RW)_3_ to traumatic wound did not significantly alter the weight gain of mice during the observation (Figure 6D), indicating a good biological compatibility of PcG_3_K_5_(RW)_3_.

### Biosafety and Stability of PcG_3_K_5_(RW)_3_ *in vivo*

There are two active components of PcG_3_K_5_(RW)_3_: Pc and synthetic peptide G_3_K_5_(RW)_3_ moiety. Pc-type compounds appear to be safe, as they have been used in close association with human. Pc has been used as a color dye for outfits and underwear in fabric industry for decades. A Pc derivative (Photosense®) has been used as an antitumor drug for cancer treatment in Ukraine since 1990s. In China, a Pc-based compound (Photocyanine®) is currently in Phase II clinical trial for patients with esophagus cancer with a dose of 0.05–0.1 mg/kg (Shao et al., 2012). Its Phase I trial demonstrated no major adverse effect in human at a dose of 2 mg/kg. Pc green (CAS Reg. No. 1328-53-6) has been approved by the FDA to use in contact lens, surgical suture, and latex condom as a color additive.

**Figure 7:**
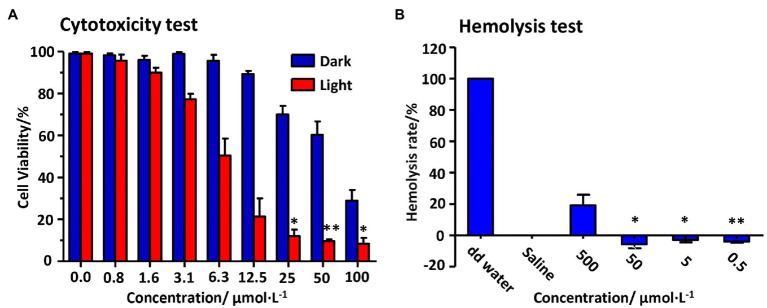
Biosafety of PcG_3_K_5_(RW)_3_ *in vivo* to human embryonic lung fibroblast (HELF) cells **(A)** with or without light illumination (12 J/cm^2^, 33.33 mW/cm^2^) and to the red blood cells **(B)**. Pure water was used as a control. Error bars indicated SD for three replications. * and ** indicated significant differences (*p* < 0.05 and *p* < 0.01, respectively) from the corresponding control group.

Despite these apparent safety records of Pc, we still evaluated the biosafety of PcG_3_K_5_(RW)_3_ experimentally. We first used the human embryonic lung fibroblast (HELF) cells and red blood cells of mice to evaluate biosafety *in vitro*. The cells were incubated with PcG_3_K_5_(RW)_3_ for 24 h, and the viability was measured by MTT assay (Figure 7A). The result showed that over 90% of cells survived after incubation with the medium concentration (IC_50_ concentration against *S. aureus* in the absence of light illumination) of PcG_3_K_5_(RW)_3_. Under illumination of 12 J/cm^2^, the peptide killed only 5% of HELF cells at the concentration of 0.8 μM while inhibiting more than 99.9% of *S. aureus* or 99% of *E. coli*, demonstrating low cytotoxicity to normal mammalian cells. In addition, PcG_3_K_5_(RW)_3_ exhibited no cytotoxicity to red blood cells at concentrations of 50 μM (Figure 7B), while IC_50_s of peptide against bacterial strains are below 25 μM.

**Figure 8:**
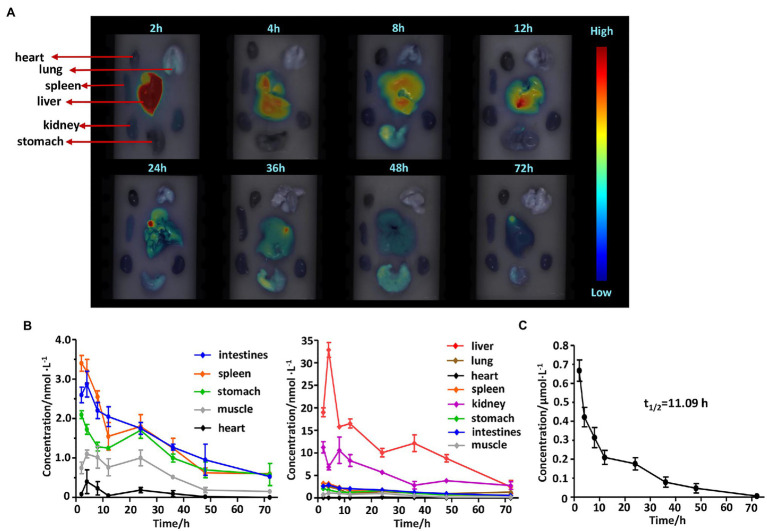
Biodistribution and clearance of PcG_3_K_5_(RW)_3_ in mice. **(A)** *Ex vivo* fluorescence images of resected organs and tissues from the mice at different time points after injection (2–24 h post-injection). **(B)** Concentration of PcG_3_K_5_(RW)_3_ in various organs and tissues at different time points after injection in 72 h after intravenous injection. **(C)** The concentration of PcG_3_K_5_(RW)_3_ in mouse blood at different time points after intravenous injection. Error bars indicated SD for three replications.

Biodistribution and clearance of PcG_3_K_5_(RW)_3_ in organs/tissues were also studied in mice. As shown in Figure 8A, PcG_3_K_5_(RW)_3_ accumulated mainly in livers and kidneys but much less in other organs/tissues. The average concentrations of PcG_3_K_5_(RW)_3_ in the livers and kidneys reached ~35 nM and ~12 nM at 2–4 h (Figure 8B) and reduced gradually with time. At 72 h post-injection, the peptide average concentrations in primary organs/tissues were reduced to low levels (2–3 nM in livers and kidneys and lower than 1 nM in other organs/tissues). The results showed that PcG_3_K_5_(RW)_3_ are mainly accumulated in liver and lung and cleared out in 72 h. Monitoring the Pc fluorescence intensity of compound **4** in mouse blood circulation at different time points after intravenous injection showed the plasma half-life of compound **4** was 11.09 h (Figure 8C).

## Data Availability Statement

The raw data supporting the conclusions of this article will be made available by the authors, without undue reservation.

## Ethics Statement

The animal study was reviewed and approved by Institutional Animal Care and Use Committee.

## Author Contributions

MH designed the project. CY and MH led the project and finalized the manuscript. DZ and QJ carried out the experiments, analyzed the results, and wrote the draft. JC, ZC, AU, LJ, and KZ assisted in the experiments and result analysis. All authors contributed to the article and approved the submitted version.

### Conflict of Interest

The authors declare that the research was conducted in the absence of any commercial or financial relationships that could be construed as a potential conflict of interest.
