# Supplementary material for: Development of a Potent Antimicrobial Peptide With Photodynamic Activity
Source: Front Microbiol. 2021 Jun 1;12:624465. doi: 10.3389/fmicb.2021.624465 (PMC8203924; doi:10.3389/fmicb.2021.624465)
Supplement: Supplementary file 1 [file Data_Sheet_1.DOCX]

Supplementary Material

## Supplementary Figures


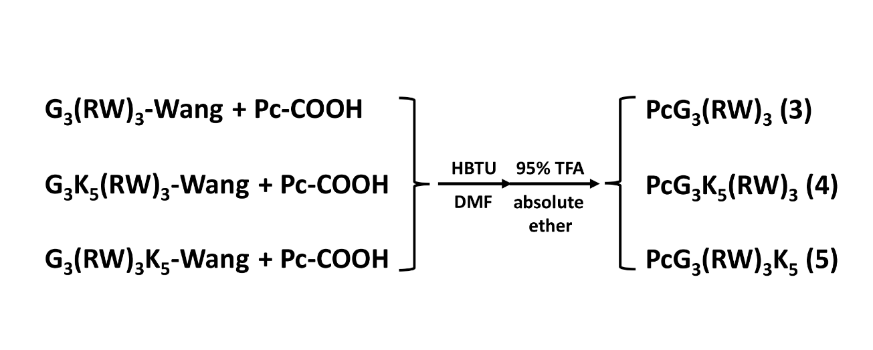


**Figure S1.** Synthesis procedure of new antimicrobial peptides


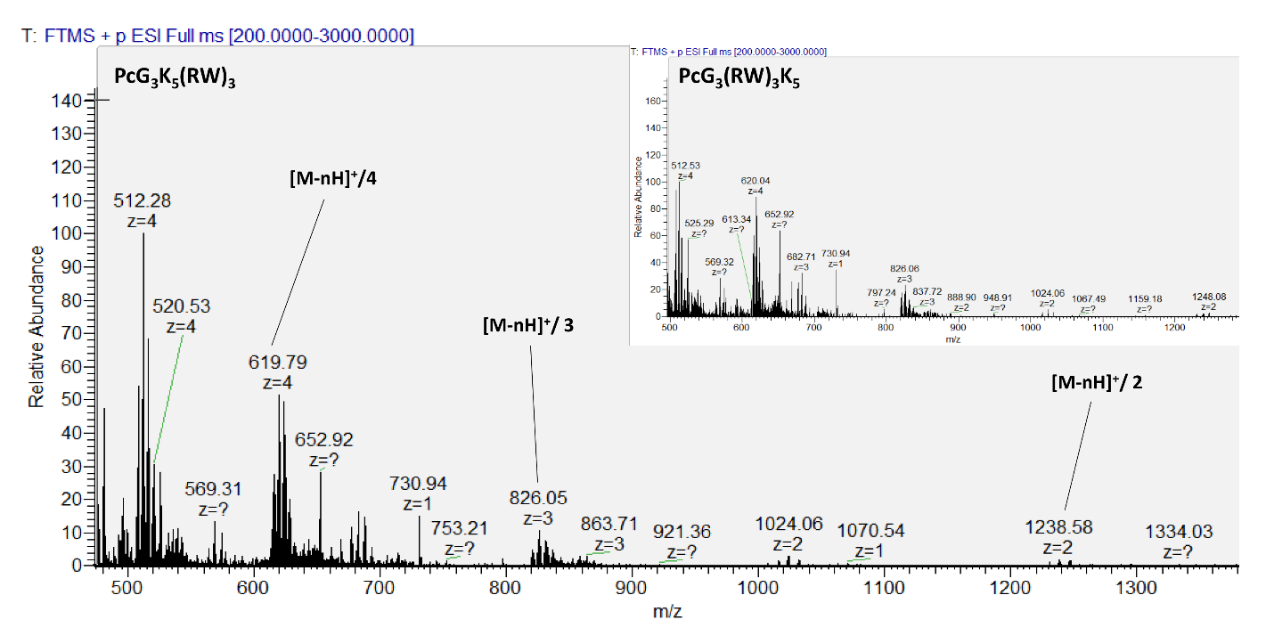


**Figure S2.** ESI-MS spectrum of hydrosoluble APPs.


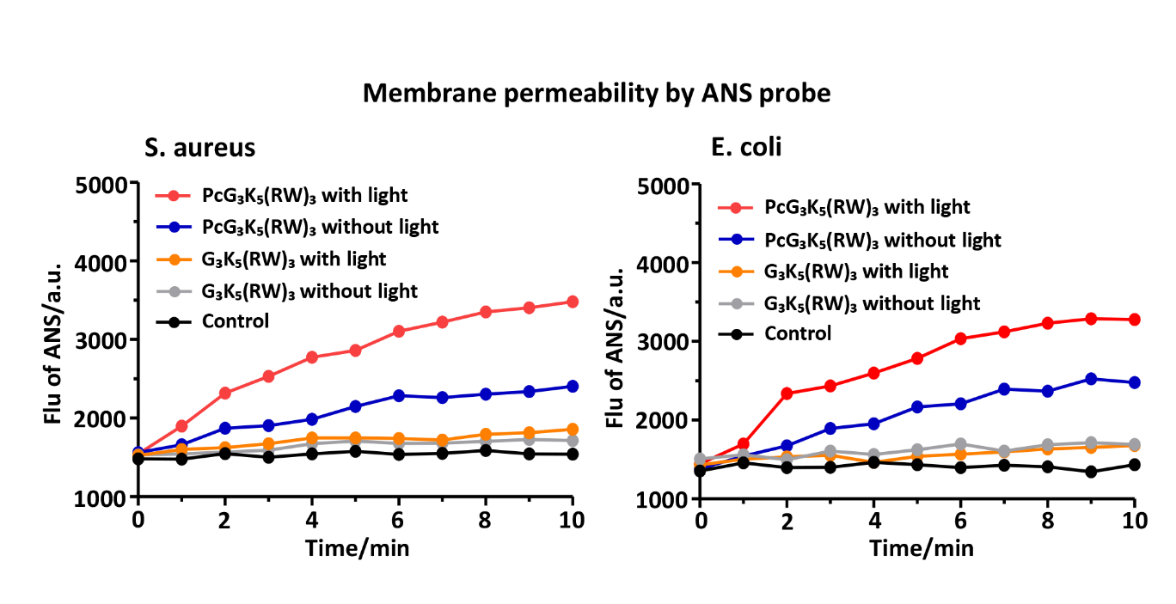


**Figure S3****.** New peptides affected bacterial membrane permeability as measured by a fluorescence probe ANS. Bacterial suspension was treated with peptides (final concentration of 10 µM) with or without light (12 J/cm^2^), and then the probe ANS was added to the suspension. The fluorescence intensity recorded reveals superiority of APPs on antimicrobial effect, and enhancement from illumination to activity. Each experiment was performed in triplicate


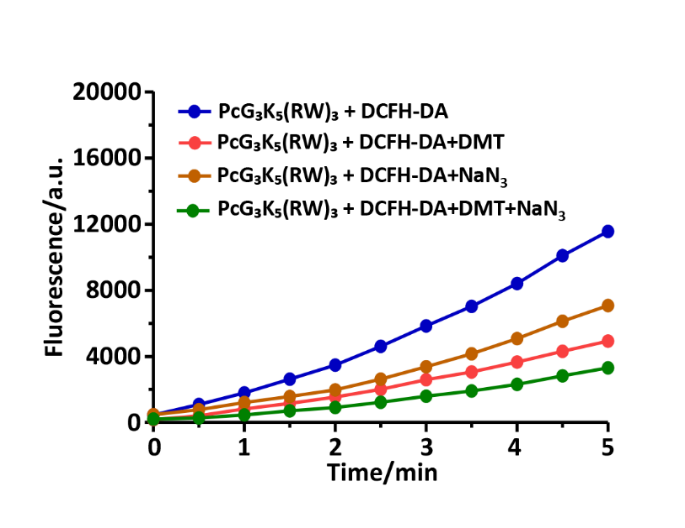


**Figure S4.** Measurements of reactive oxygen species (ROS) generated by PcG₃K₅(RW)₃ **(4)**. Fluorescence of the ROS probe DCFH-DA (100 μM, ex 400 nm, em 528 nm) in the presence of PcG₃K₅(RW)₃ (10 μM in PBS), and the effects of DMT (1 M) and/or NaN3 (30 mM). Each experiment was performed in triplicate


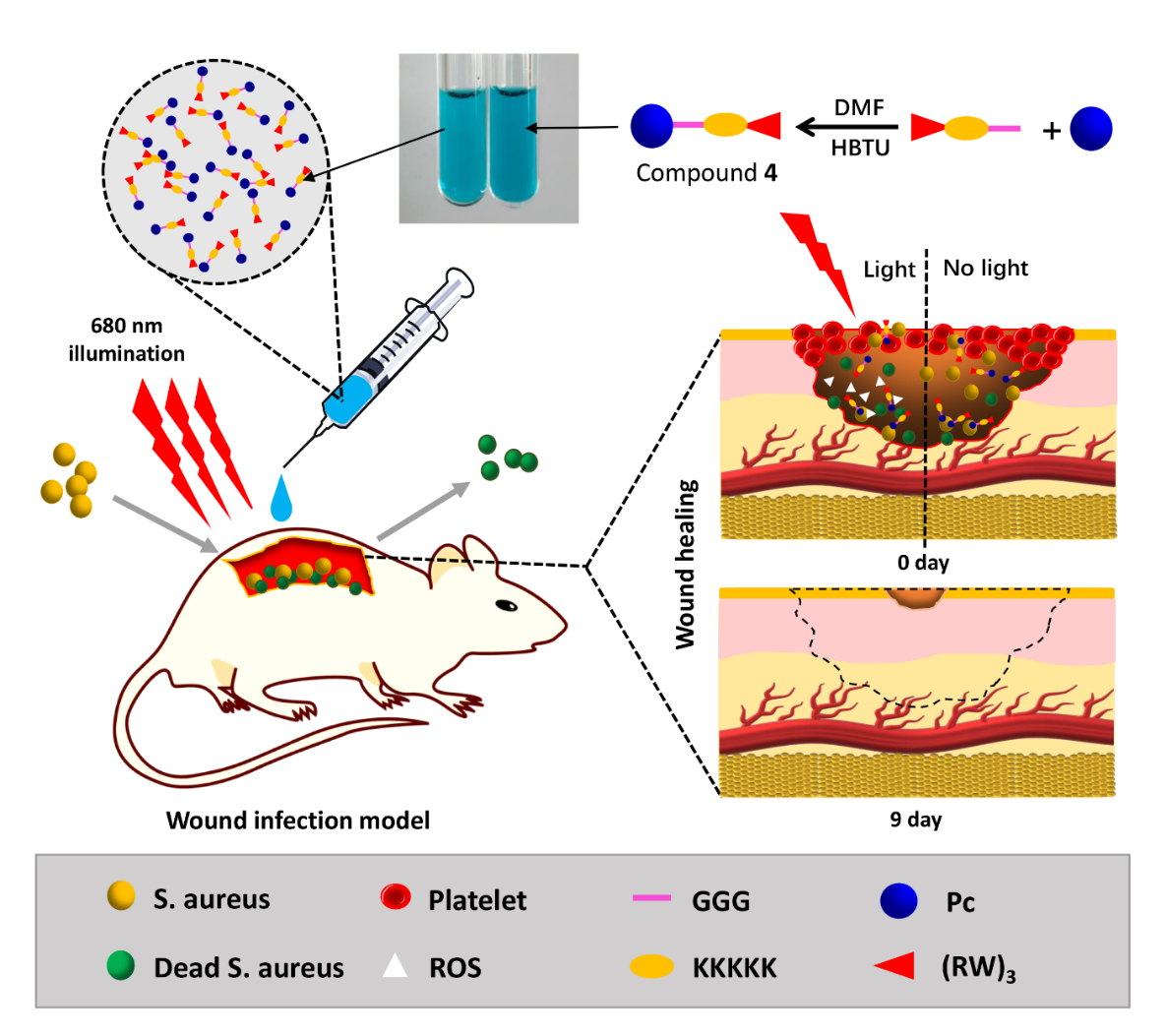


**Figure S5.** Outline of wound infection mice model under light illumination.
